# Le kyste hydatique du cordon spermatique: une localisation exceptionnelle

**Published:** 2011-12-18

**Authors:** Mohamed Moncef Hamdane, Fethi Bougrine, Issam Msakni, Amen Dhaoui-Ghozzi, Ammar Bouziani

**Affiliations:** 1 Service d'Anatomie Pathologique Hôpital Militaire Principal d'Instruction de Tunis, Montfleury, 1008-Tunis,Tunisie

**Keywords:** Hydatidose, cordon spermatique, urologie, anatomie pathologique, Tunisie

## Abstract

L’ hydatidose est une anthropo-zoonose due au développement chez l'homme de la forme larvaire du taenia *Echinococcus granulosis*. La plupart des kystes hydatiques se localisent dans le foie et les poumons. Le kyste hydatique du cordon spermatique est extrêmement rare avec seulement 4 cas rapportés dans la littérature. Les auteurs rapportent dans cet article un nouveau cas d'hydatidose du cordon spermatique. Il s'agissait d'un homme de 40 ans qui consultait pour des douleurs scrotales évoluant depuis huit mois. L'examen clinique a mis en évidence une tuméfaction mobile, inguino-scrotale, droite. L’échographie testiculaire a objectivé une hernie inguinale droite associée à deux kystes épididymaires bilatéraux. Le patient a été opéré pour cure de son hernie avec découverte en per-opératoire d'un kyste du cordon spermatique qui a été réséqué. L'examen anatomopathologique a conclu à une hydatidose du cordon spermatique.

## Introduction

L’échinococcose est une parasitose cosmopolite, endémique en Tunisie, due au développement chez l'homme de la forme larvaire *d'Echinococcus granulosus*, petit tænia vivant dans l'intestin du chien. Le foie et les poumons sont de loin les organes les plus touchés par la parasitose [1]. Le kyste hydatique du cordon spermatique (KHCS) est exceptionnel avec seulement 4 cas rapportés dans la littérature médicale [2–5].

## Observation

**Figure 1:**
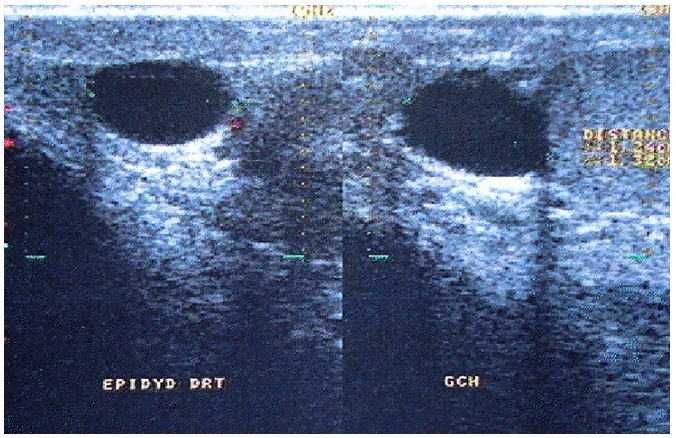
Echographie testiculaire montrant des kystes épididymaires bilatéraux, sans paroi propre

Nous rapportons le cas d'un homme âgé de 40 ans, sans antécédents notables, exploré pour douleurs scrotales droites évoluant depuis 8 mois. L'examen clinique a objectivé une tuméfaction inguino-scrotale droite, mobile, sans fièvre ni signes urinaires ou troubles de transit. L’échographie testiculaire a conclu à une hernie inguinale droite dont le collet mesurait 18 mm. Ils s'y associaient deux kystes épididymaires bilatéraux, sans paroi propre (Figure 1), ainsi qu'une varicocèle gauche. Le testicule gauche était, par ailleurs, hypotrophique.

**Figure 2:**
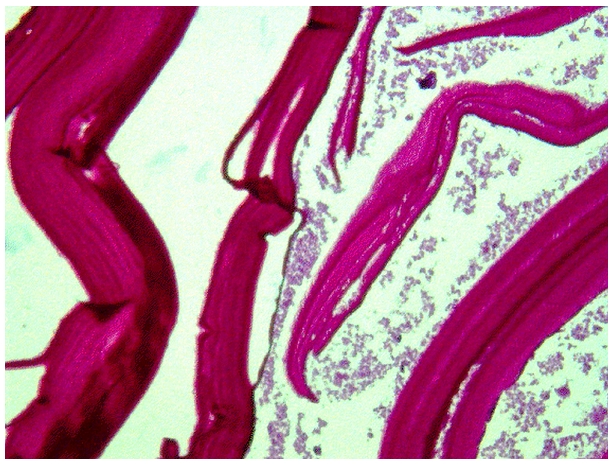
Cuticule: membranes éosinophiles, anhistes, lamellaires (HEx100)

Le patient a été opéré pour cure de son hernie. L'exploration per-opératoire a mis en évidence une formation kystique se développant aux dépens du cordon spermatique. La lésion a été réséquée et adressée pour étude anatomopathologique. L'examen macroscopique a montré un kyste à paroi calcifiée, mesurant 3x2x1,5 cm. Histologiquement, il était tapissé par une paroi anhiste, éosinophile, feuilletée, PAS positive et comportait des scolex dans sa lumière (Figure 2). Le diagnostic de kyste hydatique du cordon spermatique a été retenu. Les suites opératoires ont été simples. Le patient a été perdu de vue après sa sortie du service de chirurgie.

## Discussion

L’échinococcose est une anthropozoonose cosmopolite très fréquente en milieu rural, prédominant en Afrique du Nord, dans certains pays du pourtour du bassin méditerranéen, en Nouvelle-Zélande, en Australie, en Asie, et en Amérique [6]. En Tunisie l'hydatidose, sévit sur un mode endémique et constitue un véritable problème de santé publique avec une incidence annuelle de 15/100 000 habitants et un coût spécifique de prise en charge chirurgicale et de perte de viande animale avoisinant 1260000 dollars américains [7].

Le kyste hydatique peut siéger à n'importe quel point de l'organisme mais les localisations hépatique (50 à 70%) et pulmonaire (25 à 40%) restent les plus fréquentes [1]. Habituellement, c'est le rein qui constitue la localisation la plus commune dans le tractus urogénital, représentant 5% des formes viscérales [6]. Le KHCS est exceptionnel. Le premier cas a été décrit par Chandra et Dutt en 1951 [2]. Depuis, seulement trois cas ont été rapportés dans la littérature médicale [3–5].

Le KHCS peut survenir à n'importe quel âge, touchant l'enfant [5], l'adulte [3] ou le sujet âgé [4]. Les mécanismes expliquant l'implantation du parasite au niveau de cette localisation sont encore mal connus, mais il semble que la dissémination hématogène primitive des embryons hexacanthes soit l'hypothèse la plus plausible [4]. Cliniquement, le KHCS se manifeste par une tuméfaction inguinale, mobile, indolore, de taille variable, posant un problème de diagnostic différentiel avec les autres causes de masses inguinales, à savoir une hernie, une hydrocèle enkystée du cordon spermatique, un lymphangiome ou un kyste du cordon spermatique... [4,5]. L’échographie permet d'identifier le siège de la lésion, d'orienter vers le diagnostic de kyste hydatique et de le classer selon la classification Gharbi. Le diagnostic est cependant rarement évident, surtout pour les KHCS de stade I qui sont difficiles à distinguer d'un kyste simple du cordon spermatique ou d'une hydrocèle [4]. Les KHCS de stade IV sont aussi de diagnostic difficile à cause de leur aspect pseudo-tumoral, posant le problème de diagnostic différentiel avec les autres néoplasies du cordon spermatique [4].

Le diagnostic de KHCS est souvent suspecté en per-opératoire mais n'est confirmé qu'après examen anatomopathologique [4,5]. L'examen macroscopique montre habituellement une formation kystique de taille variable, allant de 3 à 10 cm de grand axe [4,5]. A la coupe, la paroi interne du kyste est tapissée par une membrane blanchâtre, translucide. La lumière contient un liquide clair et des vésicules de différentes tailles, rondes et fragiles [1]. A l'examen microscopique, le kyste a deux membranes accolées l'une à l'autre. La membrane externe ou cuticule est formée de lamelles concentriques, stratifiées et anhistes, PAS positives. La membrane interne ou proligère, souvent difficile à voir, répond à un fin syncytium plasmodial très riche en noyaux cellulaires, à partir duquel se constituent les scolex que l′on peut parfois retrouver dans la cavité kystique. En périphérie, le kyste est entouré d'une coque ou adventice, non-parasitaire, faite d'un tissu fibro-conjonctif, riche en néo-vaisseaux [1,8].

Le traitement du KHCS est chirurgical. Il doit être conservateur en préservant la vascularisation testiculaire et en gardant un canal déférent fonctionnel. En l'absence d'adhérences avec les éléments nobles (vaisseaux spermatiques, canal déférent), la périkystectomie doit être totale [4]. La recherche et la cure d'autres localisations de l’échinococcose s'imposent aussi.

La surveillance clinique, biologique et radiologique régulière pendant de nombreuses années est nécessaire, afin de dépister précocement toute récidive ou apparition secondaire d'autres localisations. Cette surveillance comporte un examen clinique complet, trimestriel la première année, semestriel pendant deux ans, puis annuel pendant dix ans. De même, sont pratiquées une sérologie hydatique, une radiographie du thorax et une échographie abdominale et hépatique [4].

## Conclusion

Le KHCS est une lésion exceptionnelle qui est rarement diagnostiquée en préopératoire. Cependant, dans un pays endémique comme la Tunisie, cette entité devrait être toujours considérée parmi les hypothèses diagnostiques, devant toute lésion kystique du cordon spermatique, afin d'assurer une prise en charge adéquate et dans les meilleurs délais.
